# Modelling the Radial Growth of *Geotrichum candidum*: Effects of Temperature and Water Activity

**DOI:** 10.3390/microorganisms9030532

**Published:** 2021-03-05

**Authors:** Martina Koňuchová, Ľubomír Valík

**Affiliations:** Department of Nutrition and Food Quality Assessment, Institute of Food Science and Nutrition, Faculty of Chemical and Food Technology, Slovak University of Technology in Bratislava, Radlinskeho 9, SK-812 37 Bratislava, Slovakia; lubomir.valik@stuba.sk

**Keywords:** *Geotrichum candidum*, surface growth modelling, cardinal model, spoilage

## Abstract

Modelling the growth of microorganisms in relation to environmental factors provides quantitative knowledge that can be used to predict their behaviour in foods. For this reason, the effects of temperature and water activity (*a_w_*) adjusted with NaCl on the surface growth of two isolates and one culture strain of *Geotrichum candidum* were studied. A dataset of growth parameters obtained from almost 600 growth curves was employed for secondary modelling with cardinal models (CMs). The theoretical minimal temperature resulting from the modelling of the mycelium proliferation rate ranged from −5.2 to −0.4 °C. Optimal and maximal temperatures were calculated and found to have narrow ranges of 25.4 to 28.0 °C and 34.2 to 37.6 °C, respectively. Cardinal *a_w_* values associated with radial growth (*a_w_*_min_ from 0.948–0.960 and *a_w_*_opt_ from 0.992–0.993) confirmed the salt sensitivity of the species. Model goodness-of-fit was evaluated by the coefficient of determination *R*^2^, which ranged from 0.954 to 0.985, and *RMSE*, which ranged from 0.28 to 0.42. Substantially higher variability accompanied the lag time for growth modelling than the radial growth rate modelling despite the square root transformation of the reciprocal lag phase data (*R*^2^ = 0.685 to 0.808). Nevertheless, the findings demonstrate that the outputs of growth modelling can be applied to the quantitative evaluation of the roles of *G. candidum* in fresh cheese spoilage as well as the ripening of Camembert-type cheeses or various artisanal cheeses. Along with validation, the interactions with lactic acid bacteria can be included to improve the predictions of *G. candidum* in the future.

## 1. Introduction

Food-associated microscopic fungi and cheese-contaminating mycobiota are generally known as agents of spoilage associated with mycotoxin production. Fungal spoilage of foods usually manifests as visible mycelium, a wide variety of metabolic by-products that cause off-odours and flavours and visible changes in colour or texture [1].

Species associated with the deterioration of dairy products in the agro-food industry include *Geotrichum* species [2,3,4]. *Geotrichum candidum* can contaminate processed foods and adversely affect their nutritional quality through physical, chemical, and sensorial changes. Having mouldy and yeasty properties, *G. candidum* significantly affects the flavour, aroma, colour and texture of fresh cheeses, butter, fermented milk, cream, poultry meat, fruit juices and vegetables [4,5,6,7,8].

However, another main aspect of *G. candidum* presence in foods is considered. This microscopic fungus plays positive roles in certain dairy products as it is involved in the fermentation and ripening of both industrially and artisanally produced cheeses, thus contributing to their final characteristics. For example, in some Camembert types and various Slovakian soft cheeses (ewes’ lump and bryndza), *G. candidum* strains exist in commensal relationships with other fermentation or ripening microbiota. *G. candidum* assimilates lactic acid, increases pH and enables the growth of other surface microorganisms. In Camembert-type cheese, it undergoes mycelial growth, thus supporting *Penicillium camemberti*, protects food surfaces against contamination and produces enzymes, all of which contribute to proper ripening [9,10]. *G. candidum* is frequently used during the maturation and flavouring of mould-ripened cheeses (Camembert, Brie, St. Marcellin), blue-veined cheeses (Danablu, Roquefort, Stilton, Gorgonzola) and smear-ripened cheeses (Limburger, Münster, Livarot, Tilsit, Reblochon, Pont-l’Évêque cheese) [6,11,12,13,14,15]. In the Scandinavian drink Viili, it is one of the components of the starter culture used for the fermentation of this dairy product [16]. Moreover, *G. candidum* can be found at the early stages of kefir production as it covers the kefir grain surface [17] as well as in home-made and industrial products [18].

Cheese ripening is a complex biochemical process and for *G. candidum*, it is desirable that it only participates to some extent and contributes to commensal growth and metabolic equilibrium relationships with other members of the cheese microbiota. Fungi exhibit tolerance to a wide range of pH values; the principal factors affecting fungi development are water activity (*a_w_*) and temperature [19,20]. Therefore, we aimed to quantify the surface growth of *G. candidum* as influenced by temperature and *a_w_* adjusted with NaCl to provide models suitable for either the prediction of microbial spoilage of fresh cheeses or the optimization of ripening processes in dairy practice.

## 2. Materials and Methods

### 2.1. Fungal Strains and Culture Conditions

Two representative food-isolated strains and one culture strain of *G. candidum* were selected from a set of 18 isolates and 6 culture collection strains that had been evaluated with respect to growth rate variability in a previous study by Koňuchová and Valík [21]. Strains *G. candidum* G and *G. candidum* I, belonging to the collection of the Institute of Food Science and Nutrition (Slovak University of Technology in Bratislava, Slovakia), were isolated from cottage cheese and artisanal ewes’ lump cheese, respectively, and *G. candidum* CBS 557.83 was obtained from the Westerdijk Fungal Biodiversity Institute (Utrecht, the Netherlands). All strains were refrigerated (5 ± 1 °C) on plate count skim milk agar (SMA; Merck, Darmstadt, Germany) slants and periodically cultured in diluted SMA agar. For long-term storage, the cultures were frozen at −70 °C in tubes containing yeast malt broth (Sigma-Aldrich, St. Louis, MO, USA) supplemented with 20% glycerol.

### 2.2. Experimental Design

A complete factorial design was set up to study the effects of NaCl and temperature. Growth trials with four replicates of each *G. candidum* strain were performed with fifty combinations of experimental conditions according to the following factor levels:

Storage temperature (°C): 6, 8, 12, 15, 18, 21, 25, 30, 34, 37

NaCl (%):                            0, 1, 3, 5, 7 (*w*/*v*)

Standard growth SMA medium, which was acidified with 10 mL/L lactic acid (Sigma-Aldrich, St. Louis, MO, USA) to pH 5.5, was used in the experiments. The *a_w_* of the medium was adjusted with 1, 3, 5 or 7% sodium chloride (Sigma-Aldrich, St. Louis, MO, USA) and measured after sterilization with Novasina LabMaster-*a_w_* (Novasina, Lachen, Switzerland). The inoculum was prepared, and the growth experiments were carried out according to Koňuchová and Valík [21].

The diameters of *G. candidum* colonies (*d*) were measured using a Vernier calliper (150 × 0.02 mm, Sinochem Jiangsu, Nanjing, China) in two orthogonal directions per plate without opening the dishes, and the final values were calculated according to previous work.

### 2.3. Model Description

#### 2.3.1. Primary Model

Colony growth data were fitted using the primary growth model of Baranyi and Roberts [22] included in the in-house Excel 365 (Microsoft, Redmond, WD, USA) Add-in package ‘DMFit’ version 3.5 (ComBase, University of Tasmania Food Safety Centre, Hobart, Australia). In total, 600 growth curves were evaluated (representing two isolates and one culture collection strain cultured in quadruplicate at ten incubation temperatures and five NaCl concentrations).

#### 2.3.2. Secondary Modelling

The cardinal model by Rosso et al. [23] describes the effects of food environmental factors on microbial growth parameters, e.g., growth rate and lag time for growth. This model is associated with the gamma concept [24] and characterized by high “goodness of fit”; moreover, it provides four parameters that all have physiological meaning [20,25]. For *G. candidum*, the following models were used for radial growth rate (*RGR*) and lag time for growth (*λ*) modelling against temperature and *a_w_*:

(1) $$RGR=\;RGR_{\mathrm{opt}}\times \;CM\left(T\right)\;\times \;CM\left(a_{w}\right)$$

(2) $$\sqrt{\frac{1}{\lambda }}=\;\sqrt{\frac{1}{\lambda_{\mathrm{opt}}}}\times \;CM\left(T\right)\;\times \;CM\left(a_{w}\right)$$

where

(3) $$CM\;=\;\frac{\left(T-T_{max}\right){\left(T-T_{min}\right)}^{2}}{\left(T_{opt}-T_{min}\right)\left[\left(T_{opt}-T_{min}\right)\left(T-T_{opt}\right)-\left(T_{opt}-T_{max}\right)\left(T_{opt}+T_{min}-2T\right)\right]}$$

(4) $$CM\left(a_{w}\right)=\;\frac{\left(a_{w}-a_{w\;max}\right){\left(a_{w}-a_{w\;min}\right)}^{2}}{\left(a_{w\;opt}-a_{w\;min}\right)\left[\left(a_{w\;opt}-a_{w\;min}\right)\left(a_{w}-a_{w\;opt}\right)-\left(a_{w\;opt}-a_{w\;max}\right)\left(a_{w\;opt}+a_{w\;min}-2a_{w}\right)\right]}$$

Based on the experimental data, the cardinal temperatures (*T*_min_, *T*_max_, *T*_opt_) and *a_w_*-values (*a_w_*_min_, *a_w_*_max_, *a_w_*_opt_) as well as optimal radial growth rate (*RGR*_opt_) and lag phase duration (*λ*) were calculated with non-linear regression using the Excel Solver tool.

#### 2.3.3. Time Required to Achieve Visible Colonies

The time values for *G. candidum* to form visible 3 mm colonies (*t*_3_; *d* = 3 mm) at specific combinations of *T* and *a_w_* were calculated using the following equation:

(5) $$t_{3}=\;\lambda +\frac{d}{RGR}$$

The CM models Equations (1) and (2) with the model parameters summarized in Tables 1 and 2 were used for *λ* and *RGR*, respectively. For the calculation of the *t*_3_-value ranges, the CM models’ coefficients were reduced or increased by the error estimated.

### 2.4. Statistical Analysis and Model Evaluation

Analysis of variance of medians was used to assess the significance of the growth conditions, colony diameter and intraspecific differences in the monitored isolates and collection strain. The results are presented as means and standard deviations. Statistical analyses were carried out using Excel.

To evaluate the goodness of fit of the predictive models, i.e., their ability to describe the observed experimental data, we used the following mathematical and statistical indices: coefficient of determination *R*^2^, root mean square error (*RMSE*), mean relative error (*%MRE*), standard error of prediction (%*SEP*) and sum of squared residuals (*RSS*). These indices were calculated as follows:

(6) $$RMSE=\;\sqrt{\frac{\sum_{i=1}^{n}{\left(Y_{obs}-Y_{pred}\right)}^{2}}{n-p}}$$

(7) $$\%MRE=\;\frac{100}{n}\left(\frac{\sum_{i=1}^{n}\left|Y_{obs}-Y_{pred}\right|}{Y_{max}-Y_{min}}\right)$$

(8) $$\%SEP=\;\frac{100}{{\bar{\mu }}_{obs}}\sqrt{\frac{\sum_{i=1}^{n}{\left(Y_{obs}-Y_{pred}\right)}^{2}}{n}}$$

(9) $$RSS=\;\sum_{k=1}^{n}{\left(Y_{obs}-Y_{pred}\right)}_{k}^{2}$$

where *Y_obs_* and *Y_pred_* are the observed and predicated *RGR* or lag time data, respectively; *n* is the number of experimental observations; and *p* is the number of model parameters [26,27].

## 3. Results and Discussion

The main advantage of the cardinal model (CM) described by Rosso et al. [23] is that it provides estimates of the cardinal values of the environmental factors affecting growth (in our case, *Τ*_min_, *T*_opt_, *T*_max_, *a_w_*_min_, *a_w_*_opt_, and *a_w_*_max_), which are not easy to determine experimentally because fungal growth can occur several months after initial incubation [28]. Moreover, the CMs were successfully used as a predictor of the lag phase duration and/or *RGR* [25,29,30,31,32,33,34,35,36,37,38]. This work is based on hundreds of growth curves and provides outputs of lag time and *RGR* from secondary modelling. In preliminary experiments, the microscopic fungus *G. candidum* did not grow on the surface of skim milk agar at 5 °C or 38 °C over a period of more than 30 days, which is in accordance with Eliskases-Lechner et al. [8]. Thus, the mycelium growth of the two tested isolates and one collection strain was monitored at temperatures from 6 to 37 °C at intervals of 2–5 °C. 

### 3.1. Primary Surface Growth Modelling

Independently of the tested factor, the growth of *G. candidum* typically followed log-linear sigmoidal curves. The high individual coefficients of determination (with average value of *R*^2^ = 0.987 ± 0.024; *CV* = 2.4%) indicated the suitability of the Baranyi model used to fit the growth curves and determine the growth parameters. Generally, slightly higher variability among *R^2^* values was observed within quadruplicates at lower values of temperature and *a_w_*. On the other hand, as expected for the growth parameters, significantly lower variability was recorded among growth rates than among lag phase durations, as has been reported by various authors [21,28,39,40,41]. The estimated values of lag phase duration (*λ*, days) and maximum *RGR* (mm/d) for the strains under all of the experimental conditions are summarized in Supplementary Material Table S1.

### 3.2. Secondary Modelling

#### 3.2.1. Combined Effects of *a_w_* Adjusted with NaCl and Temperature on Lag Time

**Figure 1:**
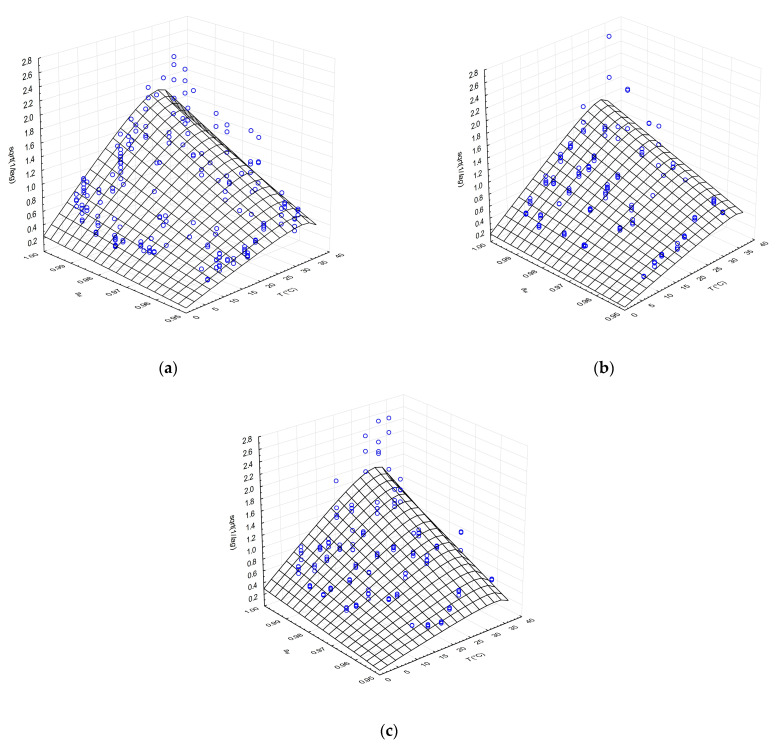
Effects of temperature and *a_w_* on the lag time (*λ*) of isolate G (**a**), isolate I (**b**) and collection strain CBS 557.83 of *G. candidum* (**c**) fitted with CM. Points (○) represent observed values of lag phase.

Despite the high variability of the lag time data, particularly in the optimal temperature range and higher temperatures approaching the maximal growth temperature, the CM with square root transformation of reciprocal lag values was able to describe the effects of *a_w_* and temperature. The outputs of lag phase modelling for the cheese-isolated and CBS culture strains showed similar patterns of behaviour, as shown in Figure 1. Lag phase decreased with increasing *a_w_* and temperature and slightly increased in the area beyond the optima, towards the maximal values of these factors (Figure 1a–c). Several qualitative radial growth studies of *Aspergillus* species, *Penicillium* species, *Fusarium oxysporum*, *Mucor circinelloides*, *Rhizopus oryzae* and *Cladosporium cladosporioides* demonstrated similar pattern of response to temperature and/or *a_w_* on lag phase duration [28,30,42,43].

All of the parameter estimates of the CM of the lag phase were consistent across the *G. candidum* strains, except for 1/*λ*_opt_, which was accompanied by higher but acceptable errors (Table 1). Similar standard deviations of CM parameters in lag time secondary modelling were reported by Marín et al. [43] for *a_w_* effect on *A. flavus* isolates and by Dagnas et al. [44] for temperature and *a_w_* effect on various bakery product spoilage moulds. 

The statistical indices of the lag phase secondary modelling revealed variability around the estimated model data, with coefficient of determination (*R*^2^) values ranging from 0.685 to 0.808. These values are acceptable considering the many influencing factors during the population adjustment period. Relative error estimates for the individual strains were calculated as *%SEP* and *%MRE* (using the range *Y*_max_–*Y*_min_ in the denominator) and ranged between 23.3 and 32.4% and from 4.9% to 9.5%, respectively.

#### 3.2.2. Combined Effects of *a_w_* and Temperature on the Growth of *G. candidum*

**Figure 2:**
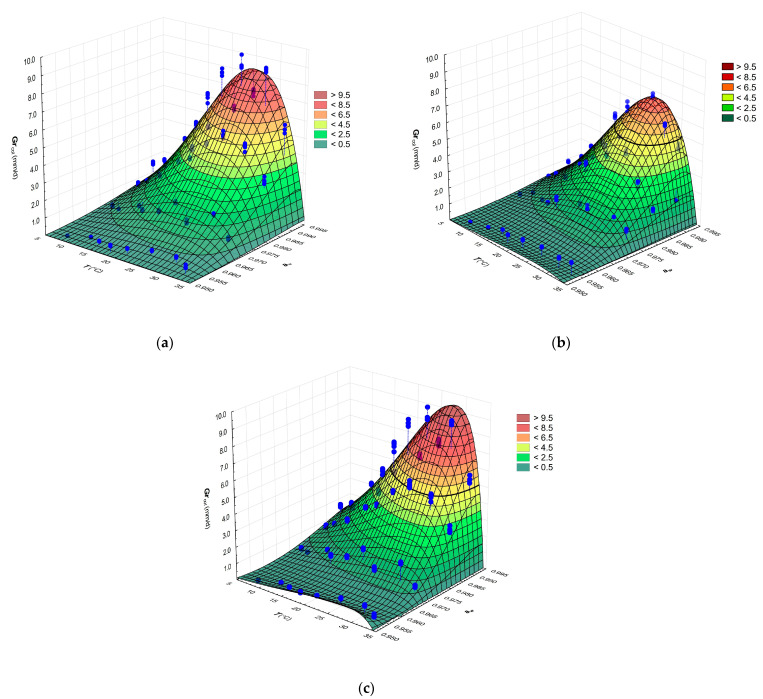
Effects of temperature and *a_w_* on the *RGR* of isolate G (**a**), isolate I (**b**) and collection strain CBS 557.83 of *G. candidum* (**c**) fitted with CM. Points (●) represent observed values of *RGR*.

The CM was used to estimate relationships between the *RGR* values of the three cultures of *G. candidum* and temperature and *a_w_* as independent variables. Figure 2a–c show that *RGR* initially increased with both increasing temperature and increasing *a_w_* and the optimal range was 25–28 °C, with maximal values between 7.85 to 9.13 mm/d and a gradual decrease beyond 10 °C. 

The CM parameter values (Table 2) and 3D surface plots indicate that the two *G. candidum* cheese isolates behaved almost identically. The collection strain CBS 577.83 showed differences from the isolates, mainly in having higher values of *T*_opt_, *T*_max_ and *RGR*_opt_. On the other hand, the mentioned collection strain was more drastically affected by reduction of *a_w_* represented by 3% of NaCl than tested isolates of *G. candidum* at 25 °C. Decreasing the *a_w_* level from a 0% to 7% NaCl content in the medium involved more than 14-fold reduction in *RGR* of collection strain *G. candidum* CBS 557.83 (Supplementary Material Table S1).

With some variation among the strains, the secondary models exhibited moderately good fit to the experimental data, as measured by the indices, e.g., *RMSE* = 0.278–0.415 and *R*^2^ = 0.954–0.985. Several studies have observed that the statistical indices for effect of temperature and *a_w_* on fungal growth were also very similar [32,45,46].

The CM values of *T*_min_, *T*_opt_ and *T*_max_ estimated for *G. candidum* growth are consistent with published data by Hudecová et al. [47,48], Domsch et al. [49], Pitt and Hocking [1] and Šípková et al. [50] regarding only the effect of temperature. However, the *RGR*_opt_ values estimated in our study are higher than the value of 5.98 mm/d reported by Hudecová et al. [48]. The study differences in estimated growth rate can be attributed to differences in the strains, models and number of independent variables investigated. 

Regarding our cardinal *a_w_* parameters, the estimation of each (minimal, optimal, maximal) from the *RGR* data was consistent across all three strains in the study. Moreover, the *a_w_*_min_ are consistent with those of previously published works [1,48,51] that report the ability of *G. candidum* to grow well on the surface of artificial growth medium or cheese with 5% NaCl (approximately *a_w_* 0.97). Plaza et al. [52] observed growth of *G. candidum* isolate from decayed citrus fruits at *a_w_* 0.95 (approximately 7% NaCl). However, these authors reported almost two times slower growth rates (3.5 mm/d) compared with growth of monitored *G. candidum* isolates G and I and strain CBS 557.83 at 30 °C at 0.995 *a_w_* level (unmodified *a_w_*). Other studies have also confirmed the salt sensitivity of *G. candidum*. Growth of *G. candidum* strains was slowed at 1–2% of NaCl on the surface growth medium [53,54,55]. Medium containing more than 4% [53,54] or 5–6% concentrations of salt [55] showed an inhibitory effect. These findings are in agreement with Hudecová et al. [14] and Marcellino and Benson [53] that salt sensitivity of *G. candidum* is strain dependent.

### 3.3. Prediction of the Time Required to Achieve Visible Colonies

The outputs of the growth modelling of *G. candidum* presented above can be used for several types of predictions in dairy practice. Regarding microscopic fungi, it is often of interest to estimate the time required to yield a visible (usually 3 mm) colony (*t*_3_; Table 3) under a set of specific environmental conditions for several purposes [31,42,56,57,58], such as evaluating microbial loads of contaminants that can detrimentally affect food before the “use by” date, identifying intrinsic or extrinsic factors that prevent the surface growth of mycelia on food for a certain period and determining growth/no growth zones. For such purposes, CM parameters for rapidly growing strains or typical strains could be used. In this work, which was aimed at “machinery mould” species, i.e., indicators of the efficacy of cleaning and sanitation procedures in dairies, six sets of CM parameters (representing three strains, *λ* and *RGR*) were developed and are available for prediction.

Having the lowest growth rate and the longest lag durations among the strains, strain I could be excluded from consideration, but the question of which growth phase is crucial for achieving the shortest *t*_3_ remained for the other two strains. To obtain answers, we calculated time data for all strains and conditions tested, which supported the exclusion of strain I from the evaluation. Strain CBS 557.83 needed less or almost equal time to form visible colonies than isolate G at low temperatures and higher *a_w_*, whereas *a_w_* = 0.97 favoured isolate G. Because of its shorter lag phase duration, strain G also yielded shorter or similar *t*_3_ data to the culture strain CBS 557.83 at moderate and higher temperatures (14–34 °C). The results indicated that the *t*_3_ is minimized when *G. candidum* displays higher growth rates, which are closely related to optimal temperature and *a_w_* range. Other studies have also confirmed these findings [31,42,57,58].

The differences in *t*_3_ data gradually increased at *a_w_* ≤ 0.97, with lower *t*_3_ values obtained for isolate G. However, data are available for the whole ranges of temperature and *a_w_* values (Supplementary material Figure S1). Table 3 presents the lowest *t*_3_ values for *G. candidum* that can be used for prediction in fresh dairy products in the temperature range 4–10 °C, at *a_w_* > 0.97 and at pH close to 5.5. As *G. candidum* can assimilate lactic acid and increase pH, e.g., by an average of 0.2 during the culture periods in this work, we speculate that data for a broad range of pH values are relevant.

## 4. Conclusions

The present work provides useful data for understanding the growth behaviour of *G. candidum* by considering the food environmental factors, temperature and *a_w_*. The inclusion of multiple strains of *G. candidum*, a “machinery mould”, in this study provided information on the variability of the model outputs that are essential for specific applications in food practice. Regarding surface growth, the time necessary to achieve visible colonies can be predicted for various values throughout the temperature and *a_w_* ranges. This prediction can be applied to various dairy products from pasteurized milk but is mainly applicable to artisanal or traditional soft or short-ripened cheeses produced from raw milk. The sensitivity of the strains to NaCl (lowering *a_w_*) seems to be the key element resulting from this work that can be applied to control growth of *G. candidum* at the cheese surface. Thus, for example, further experiments with controlled dry salting of cheese curd are needed to provide a validation study for cheese practice in the future. 

On the other hand, for industrial fresh cheese practice, the prediction models incorporated in the Monte Carlo simulation may assist in development of spoilage-prevention strategies and product shelf-life estimations. Taking the mould prevalence in the cheese packaging during production into account, together with input model data distributions, the probability as well as uncertainty of visible contaminated production can be quantified during the storage (or period of shelf life).

Another challenge is the application of other lactic acid bacteria starters or adjunct protection cultures and incorporating their interactions with *G. candidum* into stochastic simulations. This would be necessarily associated with the further studies on activity and design of protection LAB cultures suitable for the mould(s) control.

## Figures and Tables

**Table 1:** Estimated cardinal model (CM) parameters and statistical indices for lag time modelling of *G. candidum* against temperature and *a_w_*.

Parameters	Isolate G	Isolate I	Strain CBS 577.83
1/*λ*_opt_ (1/d)	3.30 ± 0.25	3.64 ± 0.68	2.97 ± 1.06
*T*_min_ (°C) *	−5.15 ± 0.10	−5.45 ± 0.14	−1.95 ± 0.03
*T*_opt_ (°C)	33.13 ± 0.32	34.56 ± 0.70	35.83 ± 0.94
*T*_max_ (°C)	37.35 ± 0.09	37.09 ± 0.11	37.06 ± 0.27
*a_w_*_min_	0.927 ± 0.003	0.938 ± 0.003	0.925 ± 0.003
*a_w_*_opt_	0.998 (fixed value)	0.998 (fixed value)	0.997 (fixed value)
*a_w_*_max_	0.999 (fixed value)	0.999 (fixed value)	1 (fixed value)
*RMSE*	0.268	0.280	0.173
*%MRE*	9.5	7.6	4.9
*%SEP*	32.0	32.4	23.3
*n*	187	159	152
*R* ^2^	0.707	0.685	0.808

± (standard deviation); *RMSE*—root mean square error; *%MRE*—mean relative error; *%SEP*—standard error of prediction; *n*—number of data points; *R*^2^—coefficient of determination; * *T*_min_ means only theoretical *T*_min_ value.

**Table 2:** Estimated CM parameters and statistical indices for radial growth rate (*RGR*) modelling of *G. candidum* against temperature and *a_w_*.

Parameters	Isolate G	Isolate I	Strain CBS 577.83
*RGR*_opt_ (mm/d)	7.85 ± 0.15	6.87 ± 0.15	9.13 ± 0.20
*T*_min_ (°C) *	−1.46 ± 0.01	−0.43 ± 0.01	−5.17 ± 0.05
*T*_opt_ (°C)	25.92 ± 0.04	25.41 ± 0.04	28.03 ± 0.37
*T*_max_ (°C)	35.63 ± 0.07	34.17 ± 0.38	37.57 ± 0.06
*a_w_*_min_	0.9479 ± 0.0014	0.9557 ± 0.0016	0.9591 ± 0.0012
*a_w_*_opt_	0.9934 ± 0.0003	0.9919 ± 0.0003	0.9916 ± 0.0002
*a_w_*_max_	0.9977 ± 0.0010	0.9988 ± 0.0011	0.9991 ± 0.0001
*RMSE*	0.415	0.405	0.278
*%MRE*	4.2	4.3	4.4
*%SEP*	16.4	20.0	19.7
*n*	192	184	188
*R^2^*	0.985	0.954	0.980

± (standard deviation); *RMSE*—root mean square error; *%MRE*—mean relative error; *%SEP*—standard error of prediction; *n*—number of data points; *R*^2^—coefficient of determination; * *T*_min_ means only theoretical *T*_min_ value.

**Table 3:** Comparison of the time needed for *G. candidum* to create 3 mm colonies.

Temperature (°C)	% NaCl	*t*_3_ (d)		*RMSE* for the *t*_3_ Predictions Based on Both Cultures Data (*n* = 6)
		Strain CBS 557.83	Isolate G	
4	0.995	8.1 ± 0.3	9.6 ± 0.4	0.9
	0.99	8.6 ± 0.5	10.6 ± 0.2	1.1
	0.98	13.8 ± 0.6	15.9 ± 0.05	1.2
	0.97	32.3 ± 1.9	28.9 ± 0.9	2.4
5	0.995	6.6 ± 0.3	7.4 ± 0.3	0.5
	0.99	7.1 ± 0.4	8.0 ± 0.1	0.6
	0.98	11.3 ± 0.5	12.9 ± 0.04	0.6
	0.97	26.4 ± 1.5	21.9 ± 0.7	2.8
6	0.995	5.5 ± 0.2	5.9 ± 0.2	0.3
	0.99	5.9 ± 0.3	6.5 ± 0.1	0.4
	0.98	9.4 ± 0.4	9.7 ± 0.04	0.3
	0.97	22.0 ± 1.3	17.3 ± 0.5	2.8
7	0.995	4.7 ± 0.2	4.8 ± 0.2	0.2
	0.99	5.0 ± 0.3	5.3 ± 0.1	0.2
	0.98	7.9 ± 0.4	7.9 ± 0.4	0.2
	0.97	18.6 ± 1.1	14.1 ± 0.4	2.7
8	0.995	4.0 ± 0.2	4.0 ± 0.2	0.1
	0.99	4.3 ± 0.3	4.4 ± 0.1	0.2
	0.98	6.8 ± 0.3	6.6 ± 0.04	0.2
	0.97	15.9 ± 1.0	11.7 ± 0.3	2.5
9	0.995	3.5 ± 0.1	3.4 ± 0.1	0.1
	0.99	3.7 ± 0.2	3.8 ± 0.1	0.2
	0.98	5.9 ± 0.3	5.6 ± 0.04	0.2
	0.97	13.8 ± 0.8	9.9 ± 0.3	2.3
10	0.995	3.0 ± 0.1	2.9 ± 0.1	0.1
	0.99	3.2 ± 0.2	3.3 ± 0.1	0.1
	0.98	5.2 ± 0.2	4.9 ± 0.04	0.2
	0.97	12.1 ± 0.7	8.6 ± 0.2	2.1

## Data Availability

The data presented in this study are available upon reasonable request from the corresponding author.

## Supplementary Materials

The following are available online at https://www.mdpi.com/2076-2607/9/3/532/s1, Figure S1: Effects of temperature and *a_w_* on the time needed for *G. candidum* to create 3 mm colonies, Table S1: The average surface growth parameters of the *G. candidum* isolates G and I and strain CBS 557.83 on SMA agar.
