# Supplementary material for: Modelling the Radial Growth of Geotrichum candidum: Effects of Temperature and Water Activity
Source: Microorganisms. 2021 Mar 5;9(3):532. doi: 10.3390/microorganisms9030532 (PMC7999232; doi:10.3390/microorganisms9030532)
Supplement: Supplementary file 1 [file microorganisms-09-00532-s001.zip › Supplementary Figure template.docx]

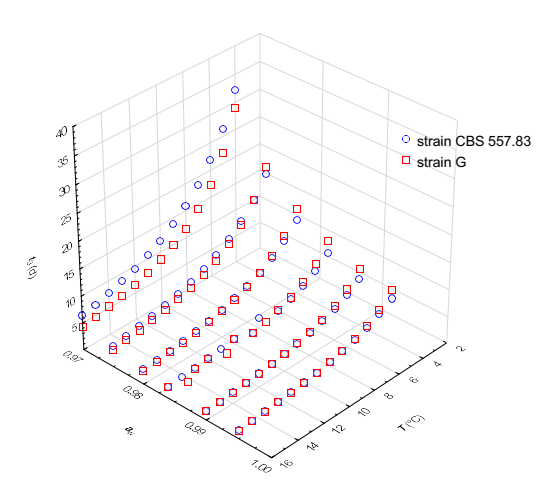


**Supplementary material Figure 1.** Effects of temperature and *a_w_* on the time needed for *G. candidum* to create 3 mm colonies.
