# Supplementary material for: Modelling the Radial Growth of Geotrichum candidum: Effects of Temperature and Water Activity
Source: Microorganisms. 2021 Mar 5;9(3):532. doi: 10.3390/microorganisms9030532 (PMC7999232; doi:10.3390/microorganisms9030532)
Supplement: Supplementary file 1 [file microorganisms-09-00532-s001.zip › Suplemmentary table template.docx]

**Supplementary material Table 1.** The average surface growth parameters of the *G. candidum* isolates G and I and strain CBS 557.83 on SMA agar.

| **Temperature**  **(°C)** | **% NaCl** | **isolate G** | | | **isolate I** | | | **strain CBS 557.83** | | |
| --- | --- | --- | --- | --- | --- | --- | --- | --- | --- | --- |
|  |  | *RGR* (mm/d) | *λ*  (d) | *d*_max_  (mm) | *RGR* (mm/d) | *λ*  (d) | *d*_max_  (mm) | *RGR* (mm/d) | *λ*  (d) | *d*_max_  (mm) |
| 6 | 0 | 1.12 | 2.02 | 55.76 | 0.85 | 5.09 | 42.47 | 1.28 | 2.60 | 65.27 |
|  | 1 | 1.29 | 0.92 | 61.25 | 1.02 | 8.55 | 41.09 | 1.72 | 5.00 | 63.01 |
|  | 3 | 0.63 | 7.42 | 39.18 | 0.57 | 14.07 | 33.54 | 0.86 | 5.30 | 45.70 |
|  | 5 | 0.12 | 6.17 | 12.99 | 0.12 | 6.91 | 10.91 | 0.21 | 5.30 | 12.90 |
|  | 7 | - | - | - | - | - | - | - | - | - |
| 8 | 0 | 1.40 | 1.21 | 65.64 | 1.12 | 3.00 | 56.37 | 1.40 | 1.58 | 60.96 |
|  | 1 | 1.66 | 3.14 | 73.47 | 1.42 | 5.90 | 58.42 | 2.00 | 2.11 | 78.77 |
|  | 3 | 1.20 | 8.30 | 50.39 | 0.97 | 10.80 | 39.76 | 1.21 | 4.43 | 52.62 |
|  | 5 | 0.29 | 9.28 | 25.21 | 0.32 | 13.10 | 22.89 | 0.25 | 5.05 | 22.22 |
|  | 7 | - | - | - | - | - | - | - | - | - |
| 12 | 0 | 2.76 | 1.48 | 76.22 | 2.36 | 1.43 | 60.69 | 2.79 | 1.36 | 74.93 |
|  | 1 | 3.24 | 2.09 | 78.96 | 2.71 | 3.30 | 59.13 | 3.53 | 1.86 | 84.35 |
|  | 3 | 2.13 | 2.95 | 44.57 | 1.35 | 3.14 | 30.31 | 1.99 | 2.98 | 58.18 |
|  | 5 | 0.63 | 8.11 | 24.38 | 0.52 | 2.87 | 22.89 | 0.69 | 3.75 | 27.35 |
|  | 7 | 0.11 | 3.94 | 5.62 | 0.12 | 9.53 | 8.0 | 0.12 | 6.19 | 11.05 |
| 15 | 0 | 3.98 | 0.98 | 90.26 | 3.24 | 1.89 | 64.23 | 4.20 | 1.36 | 94.10 |
|  | 1 | 4.42 | 0.84 | 91.04 | 3.30 | 2.13 | 78.21 | 4.63 | 1.30 | 99.07 |
|  | 3 | 2.72 | 2.50 | 83.29 | 2.51 | 3.45 | 59.17 | 2.89 | 1.98 | 79.27 |
|  | 5 | 0.89 | 5.86 | 41.06 | 0.84 | 4.84 | 41.35 | 0.98 | 4.87 | 43.41 |
|  | 7 | 0.11 | 5.84 | 8.12 | 0.29 | 8.39 | 13.77 | 0.30 | 7.76 | 15.07 |
| 18 | 0 | 5.69 | 0.65 | 93.91 | 4.32 | 0.95 | 86.06 | 5.43 | 0.53 | 94.12 |
|  | 1 | 6.06 | 0.86 | 92.54 | 5.28 | 1.20 | 87.27 | 6.49 | 1.25 | 100.92 |
|  | 3 | 3.35 | 1.01 | 68.76 | 2.90 | 1.74 | 51.72 | 3.35 | 3.18 | 77.26 |
|  | 5 | 1.14 | 4.78 | 40.55 | 1.67 | 4.14 | 41.92 | 1.08 | 5.26 | 42.65 |
|  | 7 | 0.42 | 5.81 | 20.28 | 0.51 | 5.38 | 24.59 | 0.39 | 6.63 | 18.52 |
| 21 | 0 | 5.83 | 0.47 | 91.71 | 4.78 | 0.75 | 73.34 | 5.70 | 0.55 | 92.79 |
|  | 1 | 7.09 | 0.43 | 89.21 | 6.17 | 1.17 | 77.29 | 8.13 | 0.96 | 99.89 |
|  | 3 | 4.46 | 1.32 | 51.78 | 3.68 | 1.83 | 53.81 | 4.33 | 1.67 | 72.48 |
|  | 5 | 1.45 | 3.37 | 42.14 | 1.28 | 2.99 | 29.78 | 1.78 | 2.01 | 48.46 |
|  | 7 | 0.33 | 3.56 | 12.36 | 0.52 | 3.91 | 20.88 | 0.41 | 3.74 | 17.05 |
| 25 | 0 | 6.59 | 0.24 | 90.71 | 5.73 | 0.47 | 74.04 | 6.97 | 0.16 | 91.91 |
|  | 1 | 7.43 | 0.26 | 96.81 | 7.03 | 1.16 | 76.44 | 8.72 | 0.54 | 97.94 |
|  | 3 | 4.83 | 1.13 | 68.87 | 4.20 | 1.58 | 54.79 | 5.48 | 1.46 | 77.48 |
|  | 5 | 2.10 | 1.53 | 39.89 | 1.70 | 1.53 | 38.24 | 1.21 | 1.20 | 33.07 |
|  | 7 | 0.53 | 3.08 | 17.39 | 0.54 | 2.21 | 17.53 | 0.50 | 2.34 | 16.37 |
| 30 | 0 | 6.32 | 0.03 | 79.77 | 4.36 | 0.03 | 53.09 | 7.29 | 0.03 | 84.02 |
|  | 1 | 7.28 | 0.28 | 82.36 | 6.00 | 0.28 | 79.41 | 8.94 | 0.28 | 87.26 |
|  | 3 | 4.57 | 1.50 | 79.98 | 3.26 | 1.50 | 46.54 | 4.98 | 1.50 | 80.47 |
|  | 5 | 2.40 | 2.02 | 60.47 | 1.68 | 2.02 | 42.07 | 1.98 | 2.02 | 45.03 |
|  | 7 | 0.46 | 2.26 | 15.52 | 0.62 | 2.26 | 21.80 | 0.58 | 2.26 | 21.15 |
| 33 | 0 | 2.80 | 0.07 | 40.64 | 1.31 | 0.02 | 34.79 | 3.92 | 0.18 | 58.44 |
|  | 1 | 4.53 | 0.29 | 69.69 | 1.79 | 0.26 | 28.93 | 5.59 | 0.46 | 70.02 |
|  | 3 | 3.40 | 1.44 | 49.95 | 1.84 | 0.37 | 33.22 | 3.18 | 1.27 | 45.41 |
|  | 5 | 1.34 | 1.64 | 23.95 | 1.30 | 0.95 | 21.14 | 0.85 | 0.72 | 13.26 |
|  | 7 | 0.27 | 3.02 | 8.44 | 0.74 | 3.06 | 8.52 | 0.45 | 1.86 | 8.24 |
| 37 | 0 | 0.67 | 1.50 | 4.78 | - | - | - | - | - | - |
|  | 1 | 0.54 | - | 5.38 | - | - | - | 0.25 | 2.36 | 5.15 |
|  | 3 | 0.38 | 0.47 | 8.57 | 0.01 | 0.54 | 4.84 | 0.93 | 2.63 | 20.60 |
|  | 5 | 0.33 | - | 5.42 | 0.08 | 0.12 | 5.20 | 0.22 | 0.89 | 6.01 |
|  | 7 | - | - | - | - | - | - | - | - | - |

*RGR* - radial growth rate; λ – lag time for growth; *d*_max_ – maximal diameter
